# Revealing Energy Density in Porous Carbon Supercapacitors Using Hydroquinone Sulfonic Acid as Cathodic and Alizarin Red S as Anodic Redox Electrolytes

**DOI:** 10.1002/smll.202406467

**Published:** 2024-10-07

**Authors:** Samaneh Abbasi, Farzaneh Hekmat, Saeed Shahrokhian, Mahesh Chougale, Deepak P. Dubal

**Affiliations:** ^1^ Department of Chemistry Sharif University of Technology Tehran 11155‐9516 Iran; ^2^ Department of Chemistry Shahid Beheshti University Tehran 19839‐63113 Iran; ^3^ Centre for Materials Science School of Chemistry and Physics Queensland University of Technology (QUT) 2 George Street Brisbane QLD 4000 Australia

**Keywords:** alizarin red S, hydroquinone sulfonic acid, nitrogen doped porous carbon sphere, redox electrolyte, supercapacitors

## Abstract

Exploration of innovative strategies aiming to boost energy densities of supercapacitors without sacrificing the power density and long‐term stability is of great importance. Herein, highly porous nitrogen‐doped carbon spheres (NPCS) are decorated onto the graphite sheets (GSs) through a hydrothermal route, followed by a chemical activation. The capacitive performance of the NPCS is then enhanced by hydroquinone sulfonic acid (HSQA) incorporation in both cathodic electrolyte and electrode materials. Later, NPCS are decorated with polypyrrole (PPY), in which HSQA takes a versatile role as conjugated polymer dopant and cathodic redox additive. The capacitive performance of the negative electrodes is enhanced by incorporating of alizarin red S (ARS) as anodic redox additive. Finally, PPY(HQSA)@NPCS‐GS//NPCS‐GS asymmetric supercapacitor is assembled and tested in dual redox electrolyte system containing HQSA‐cathodic and ARS‐anodic electrolytes. This device delivers a remarkable energy density of 60.37 Wh kg^−1^, which is close or even better than lead acid batteries. Thus, the present work provides a novel pathway to develop high energy supercapacitors using redox active electrolytes for next‐generation energy storage applications.

## Graphical abstract

Porous nitrogen‐doped carbon spheres (NPCSs) are directly synthesized onto the graphite sheets. The NPCSs are then decorated with hydroquinone sulfonic acid (HQSA)‐doped polypyrrole nanostructures. The electrochemical performance of the prepared carbon supercapacitors is then enhanced using HQSA with a dual role as cathodic redox electrolyte and electrode surface modifier and alizarin red as anodic redox electrolyte.

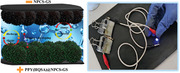

## 1 Introduction

Globally increased sustainable energy capacity in recent years, led to rising demand for high‐performance energy storage devices. Holding unique characteristics, including high power density, fast charge–discharge capability, and long‐term cyclic life supercapacitors stand out among the rest of energy storage systems.^[^ ^1^ ^]^ Notwithstanding, benefiting from favorable features, the actual implementation of supercapacitors has been hindered by their low energy densities. Correspondingly, the development of supercapacitors with high specific energies is of prime importance. While numerous research attempts have been dedicated to developing high‐performance electrodes, the role of redox electrolytes in boosting the charge storage capacity has drawn less attention.^[^ ^2^, ^3^ ^]^ Compared to conventional supercapacitors, the redox enhanced supercapacitors (RESCs) rendered an enhanced capacitive performance. This elevated performance resulted from an additional redox activity raised by the electrolytes. Charge storage in RESCs principally appears through diffusion‐controlled faradic redox reactions of the confined additives. Evidently, redox additives, which trapped into the pore space initially transformed to the adsorbed ones, then the electron transfer reaction occurred.^[^ ^4^, ^5^ ^]^ Regarding the standard redox potentials, electrochemical reversibility, toxicity, and environmental respects adding an appropriate redox additive with an optimized concentration can efficiently boost the electrochemical performance of conventional SCs.^[^ ^6^, ^7^ ^]^ The ultimate capacitive performance of the RESCs, is tightly coupled with the electrode characteristics. In this context, some important criteria containing specific surface area, porous structure, active sites, and conductivity of the prepared electrode materials should be noted. Accordingly, employing the highly porous electrodes ended in not only enhanced double‐layer capacitance (DLC) but also boosted the possibility of the redox reactions of the confined redox additives.^[^ ^8^, ^9^ ^]^

Among a wide range of porous materials, carbons are the most extensively used materials in fabrication of RESCs.^[^ ^10^, ^11^ ^]^ Taking credit from beneficial structural features, including high SSA, hierarchical porous network, short diffusion length, and remarkable electric conductivity, makes green carbons promising candidate for developing high‐performance RESCs. In other words, using highly porous structures with interconnected multiscale pores as electrode active materials ends in increased ion accessibility, therefore, accelerating ion diffusion and enhancing the rate capability of the prepared electrodes.^[^ ^12^ ^]^ Besides, the incorporation of heteroatoms into the porous carbons results in a short diffusion length that can effectively increase the wettability in parallel to raised reactive sits, and in turn, tuned electronic characteristics of the carbon material.^[^ ^13^, ^14^ ^]^

Among a variety of synthetic routes including chemical vapor deposition, molten salt assisted and arc discharge, hydrothermal carbonization (HTC), which is a scalable and eco‐friendly method, has drawn increasing attention, since it was first introduced.^[^ ^15^, ^16^, ^17^ ^]^ HTC upgrading low‐priced carbohydrates into costly chemicals. To date, tremendous research attention has been devoted to adjust the constituent, morphology and specific surface area of carbon precursors by incorporating chemical additives (e.g., NH_4_Cl, H_3_BO_3_, and ionic liquid (ILs)) within the HTC.^[^ ^16^, ^18^ ^]^ In this respect, NH_4_Cl not only promote the surface characteristics by introducing nitrogen heteroatoms into the carbonous lattice, but also improves the wettability of carbons. So, ammonium chloride participation in HTC sounds promising to promote the capacitive performance of carbon spheres.^[^ ^19^, ^20^ ^]^

Surface activation in terms of enhancing the SSA and pore spatial distribution is also of crucial significance in developing high‐performance carbon supercapacitors. Regarding the agent that is being employed, the activation approaches can be divided into three main categories: chemical, physical, and self‐activation.^[^ ^21^ ^]^ Compared to the mentioned methods, zinc chloride (ZnCl_2_)‐induced activation results in a hierarchical porous network raging from micro‐ to meso‐sized pores. Furthermore, its Lewis acid nature can further boost the dehydration of carbon precursors along with the aromatic condensation reaction. Zinc chloride‐induced activation approaches seem favorable for the preparation of well‐structured porous carbons. Accordingly, carbon sphere precursors can be successfully activated using ZnCl_2_ agent. Additionally, ZnCl_2_ thoroughly preserved the spherical morphology during the activation process.^[^ ^22^, ^23^ ^]^

Harnessing the advantage of high electric conductance and remarkable redox activity, conducting polymers (CPs) have been widely utilized for energy storage applications.^[^ ^24^ ^]^ The energy storage in CPs is associated with doping/dedoping mechanisms, where redox state conversion is accompanied by ion insertion/extraction from the polymer backbone. In synergy between two energy storage mechanisms, the co‐contribution of diffusion‐ and surface‐controlled charge storage can efficiently upgrade the capacitive performance of the CP‐decorated NPCSs. According to the literature, the delocalized charge on the backbone of the CPs can be stabilized by counter‐ion incorporation, which can effectively modify the energy levels. Doped CPs, in turn, exhibited improved conductivity compared to the undoped ones.^[^ ^25^, ^26^ ^]^ Taking advantage of adjustable electrical properties, facile variation in oxidation state, and tunable morphology, poly pyrrole (PPY) known as one of the most auspicious CPs has been widely used for energy applications.^[^ ^27^ ^]^ PPY can be directly obtained through either chemical or electrochemical polymerization. Employing the irritant and toxic oxidants alongside the probability of polymer agglomeration and lowering the electrical conductivity, the widespread application of chemical methods is considerably limited.^[^ ^28^ ^]^

Electrochemical polymerization, conversely, is a cost‐effective method, in which the thickness and functionality of the product can be easily controlled by the applied current and potential adjustment.^[^ ^29^ ^]^ Ionic dopants are spontaneously introduced to the charged chain of PPY through the electro‐polymerization approach.^[^ ^30^ ^]^ Accordingly, acids have been extensively used as p‐type oxidizing dopant in CPs polymerization, where concurrent creation of free radicals and positive charges, can be attributed to removing electrons from neutral PPY chains.^[^ ^26^ ^]^ Accordingly, the introduction of dopants into the PPY chains leads to their electrical characteristics modification. Having a profound impact on the conductance of PPYs, dopants incorporation into the polymer chain can effectively affects PPYsʹ capacitive performance. Carrying out a key role in establishing characteristic features of PPY, the adoption of redox‐active dopants, which additionally take an active role in faradic redox reactions, can further improve the specific capacitance of PPY.^[^ ^31^, ^32^ ^]^ Despite the outstanding capacitive behavior of PPY, they deprive of long‐term durability caused by the polymer swelling/shrinking within the doping/dedoping process. Stable carbonaceous materials significantly prevent the structural breakdown of PPY, so preparation of PPY/carbon composite is highly beneficial for fabrication of high performance RESCs.^[^ ^33^ ^]^ Whereas hydroquinone derivatives were utilized as either CPs modifiers or redox‐active electrolytes, their dual functionality in enhancing the capacitive performance has been rarely studied.^[^ ^34^ ^]^ In parallel to asymmetric supercapacitor electrodes, utilizing dual redox‐active electrolytes constructed from two dissimilar redox active species, in which each individual is active in either a positive or negative potential range, sounds like a feasible policy to attain a superior energy storage performance.^[^ ^35^ ^]^ Herein, binder‐free NPCS‐GS electrodes were fabricated using an innovative green strategy to upgrade low‐cost monosaccharide into high‐performance electrode active materials. Accordingly, NPCSs were directly grown onto the GSs through HTC within aqueous glucose solution, including ammonium chloride. The wet chemical HTC followed by zinc chloride (ZnCl_2_)‐induced chemical activation. HQSA‐doped PPY nanostructures were next decorated onto the NPCS‐GSs via a galvanostatic electropolymerization route. The capacitive behavior of PPY(HQSA)@NPCS‐GSs was further promoted when HQSA was also utilized as a cathodic redox additive. In cooperation with cathodic HQSA redox spices, the addition of multielectronic ARS boosted the capacitive performance of the fabricated PPY(HQSA)@NPCS‐GS//NPCS‐GS asymmetric hybrid supercapacitors. Compared to HQSA redox additives, which are active in a positive range of potential, ARS counterparts are best suited to a negative range of potential. Accordingly, the HQSA‐ARS dual redox electrolytes can simultaneously boost the capacitive performance of positive and negative electrode. This can effectively enhance the energy density of the prepared energy storage devices. The capacitive performance of asymmetric PPY(HQSA)@NPCS‐GS//NPCS‐GS devices, storing charge via both energy storage mechanisms, was stepped‐up using dual redox electrolytes. The enhanced electrochemical behavior along with their eco‐benign and low‐cost carbonization procedure, makes PPY(HQSA)@NPCS‐GS//NPCS‐GS redox enhanced asymmetric supercapacitor (REASC) immensely promising for next‐generation energy applications.

## 2 Results and Discussion

### 2.1 Morphological and Structural Investigation of the Prepared Materials

**Scheme 1:**
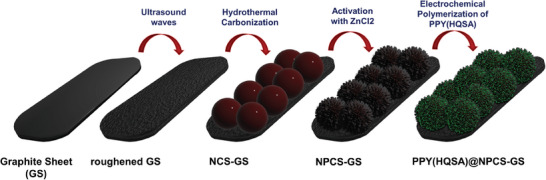
Schematic illustration of PPY(HQSA)@NPCS‐GS preparation procedure.

As it was depicted in **Scheme** **1**, NPCS uniformly grown on GS because of HTC followed by ZnCl_2_ induced activation, afterward electrochemical polymerization of pyrrole in the presence of HQSA led to the PPY(HQSA) decorated NPCS, which was employed as the positive electrode in fabrication of the REASC.

**Figure 1:**
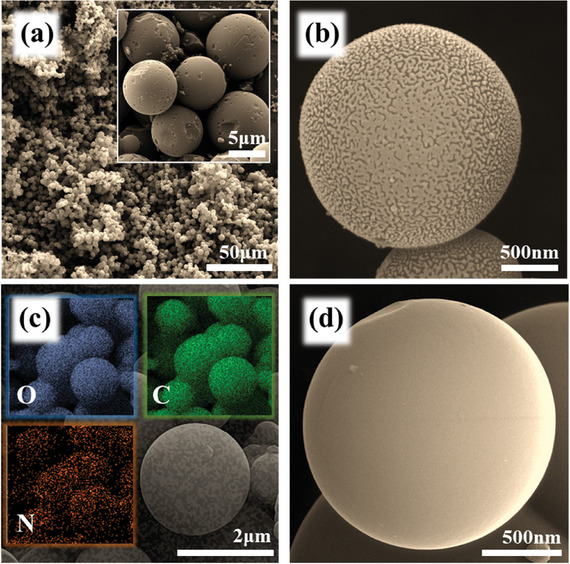
SEM images of a,b) NPCS‐GS. c) Its corresponding EDS maps in comparison to d) SEM image of NCS.

FE‐SEM images of NPCS grown directly onto GSs, are provided in **Figure** **1**. As seen in the figure, HTC of ammonium‐included glucose solution resulted in integral growth of NPCSs onto the GSs. As stated in Figure 1a, the GCs were entirely covered by highly porous carbon spheres. The magnified image of synthesized NPCSs was further presented in Figure 1b. For further comparison, a high‐magnification image of N‐doped CSs is provided in Figure 1d. As reflected in the figures, the interconnected cavities were satisfactorily formed within the surface of spherical carbons through chemical activation. Besides, by contrast to other harsh activation agents, the spherical shape of carbons was well‐preserved within activating by zinc chloride. The proper incorporation of nitrogen heteroatoms into the carbonaceous network was further confirmed considering the energy dispersive X‐ray spectroscopy (EDS) results (Figure 1c; and Figure S1, Supporting Information).

**Figure 2:**
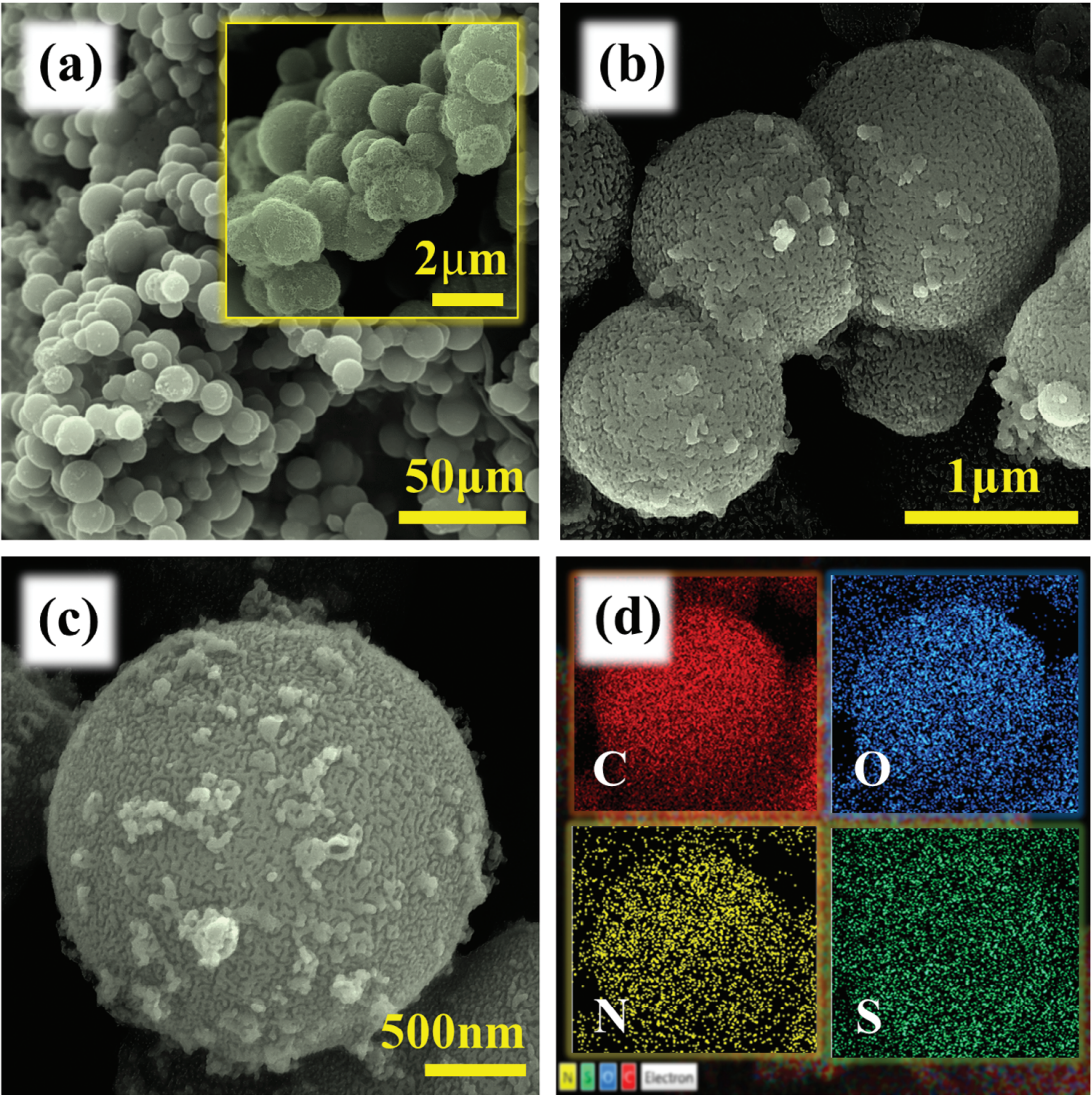
a–c) SEM images of PPY(HQSA)@NPCS‐GSs at different magnifications and d) their corresponding EDS elemental maps.

Surface morphology and elemental composition of the binder‐free PPY(HQSA)@NPCS‐GS are provided in **Figure** **2**. As seen in the figure, HQSA in coexisting with pyrrole monomers the uniform decoration of NPCS‐GS by PPY(HQSA) through galvanostatic polymerization. Accordingly, core–shell arrangements of PPY(HQSA)@NPCS‐GSs were well developed herein. Additionally, the occurrence of HQSA doping into the PPY backbone can be validated by observation of sufficient elemental sulfur in EDS mapping results (Figure 2d; Figure S2, Supporting Information). SEM images of PPY(HQSA)@NPCS‐GS after 3000 charge/discharge also provided in Figure S3 (Supporting Information) to ensure the moderate stability of prepared composite structure during charge–discharge cycles.

**Figure 3:**
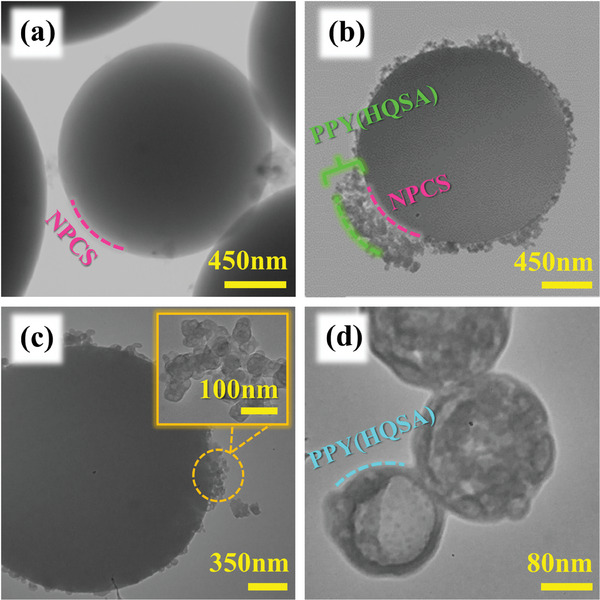
TEM images of a) NPCS. b,c) PPY(HQSA) decorated counterparts, inset: its corresponding high magnification view, and d) highly magnified PPY(HQSA) nanostructures.

The microstructure of the prepared samples is provided in **Figure** **3**. When the tranmission electrom microscopy (TEM) images of NPCSs (Figure 3a) and PPY(HQSA)‐decorated counterparts (Figure 3b,c) are compared, it is quite clear that NPCSs were integrally covered by PPY(HQSA) nanostructures. The surface morphology of the HQSA‐doped PPYs was further investigated via TEM measurements. As provided in the inset of Figure 3c,d HQSA‐doped PPYs emerged as spherical nanostructures.

**Figure 4:**
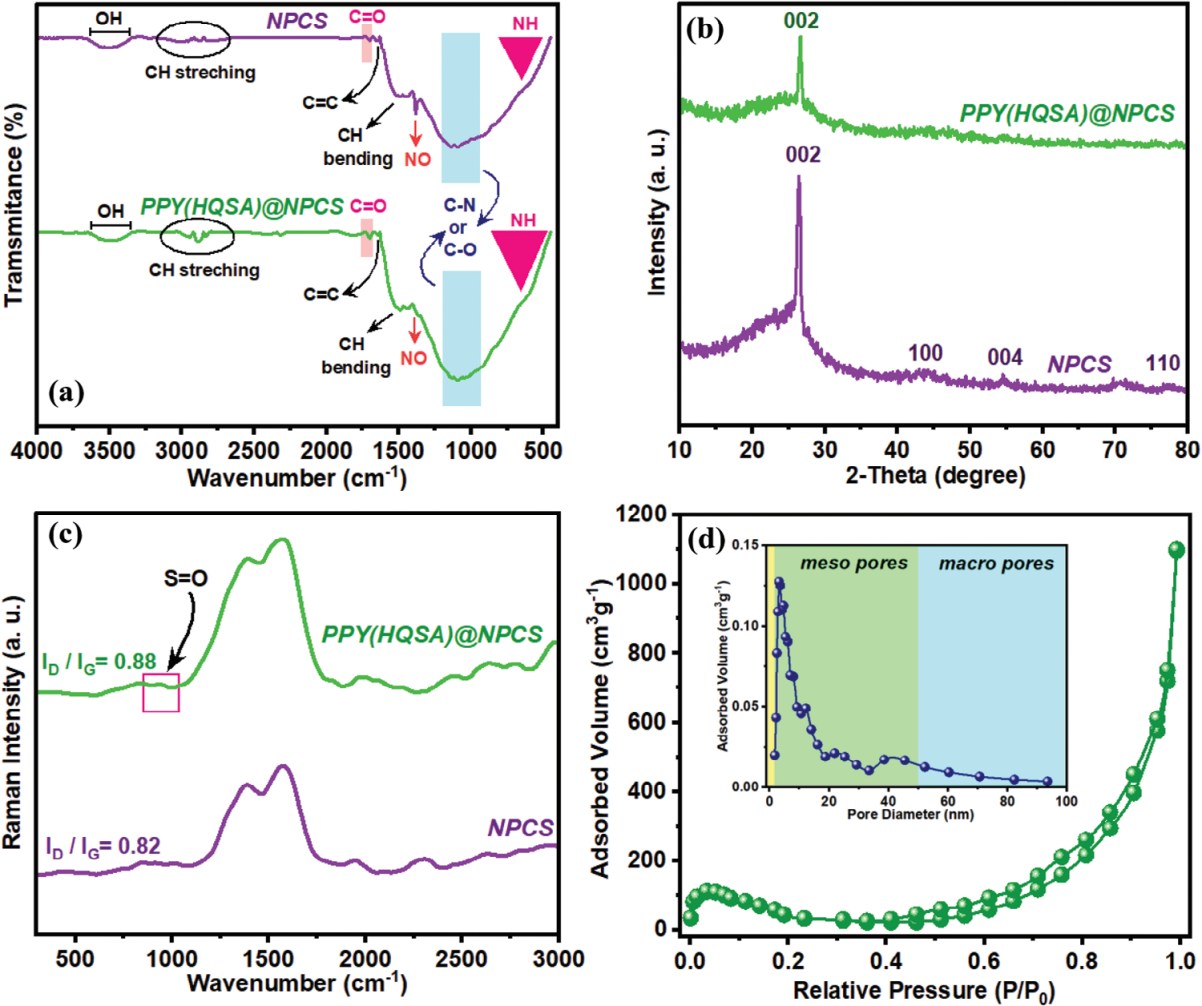
a–c) FT‐IR, XRD, and Raman spectra of NPCSs compared to PPY(HQSA)@NPCSs, and d) nitrogen adsorption/desorption isotherm of PPY(HQSA)@NPCS, inset: corresponding BJH pore size distribution.

The structural features of the NPCSs and PPY(HQSA)‐decorated NPCSs were also investigated by Fourier‐transform infrared spectroscopy (FT‐IR). As seen in **Figure** **4**a, two broad bands that appeared in 500–800, 3300 to 3700 cm^−1^ are attributed to the NH wagging and OH stretching vibrations, respectively.^[^ ^30^, ^35^ ^]^ Observation of multiple peaks at 1460–1490 cm^−1^ ascribed to CH bending modes. Characteristic peaks located at 2800–3000 cm^−1^ were generated due to the CH stretching vibrations.^[^ ^35^ ^]^ In addition, characteristic peaks of C═O vibrations emerged at 1073 cm^−1^. The corresponding peak of C═C group in NPCS is observed at 1646 cm^−1^, which slightly shifts to 1640 cm^−1^ in consequence of PPY deposition. This blueshift implies the interaction between PPY(HQSA) backbone and NPCS.^[^ ^36^ ^]^ Additionally, the observance of a broadband at 1089 cm^−1^ can be assigned to C*─*N and C*─*O vibrational modes.^[^ ^37^ ^]^ As seen in the NPCS spectrum, the observation of a peak at ≈1384 cm^−1^ can be attributed to the NO asymmetric stretching vibrations, which confirms the successful formation of nitrogen‐doped PCS. In comparison to NPCSs, the characteristic peak of nitro groups in PPY(HQSA) decorated NPCSs is shifted to 1366 cm^−1^. This significant blueshift suggests hydrogen bonding interaction between the PPY chain and nitro groups of NPCS.^[^ ^38^ ^]^ Besides, the board and strong bands of C*─*N and C*─*O vibrations covered the S═O stretching modes.^[^ ^39^ ^]^ Eventually, FT‐IR investigations present strong evidence for PPY deposition onto the NPCS‐GSs.

Both NPCS and PPY(HQSA)‐decorated NPCSs were further characterized by employing an X‐ray diffraction (XRD) spectrometer and the resulting XPD patterns are provided in Figure 4b. As seen in the figure, the observance of two characteristic peaks in NPCSs’ XRD spectrum at 2θ values of 26.43° and 54.5° can be ascribed to the (002) and (004) lattice planes of hexagonal graphite (JCPDS, card no. 00‐008‐0415).^[^ ^40^ ^]^ The well‐crystalized structure of NPCSs was evident by the appearance of a sharp (002) diffraction peak at 26.43°. An ideal interlayer space of 002 (0.3368 nm), estimated according to Bragg's law, provides further convincing evidence of the well‐crystallized structure of NPCSs.^[^ ^41^ ^]^ Upon comparing to the XRD pattern of bare NPCSs, PPY (HQSA)‐decorated counterparts are deprived of well‐resolved XRD patterns. This can be attributed to the amorphous nature of the synthesized PPY(HQSA) films. Besides, the decoration of NPCSs with amorphous PPY(HQSA) nanostructures also lowered the crystallinity of the carbon backbones, which is evidently reflected in their corresponding XRD spectrum.

Raman spectroscopy as a valuable technique for carbons and their derivatives characterization was further used to evaluate the prepared electrodes. The Raman spectra of bare and PPY(HQSA)‐decorated NPCS‐GCs are presented in Figure 4c. Two characteristic peaks of carbons came into view around 1390 cm^−1^ (D band) and 1560 cm^−1^ (G band) in both spectra. The G band represents the perfect graphitic lattice originates from E2g vibrational mode, while D band ascribed to the vibrational mode of A1g that detected in disordered graphite.^[^ ^42^, ^43^ ^]^ Consequently, the intensity ratio of D to G band reflected the crystal defects quantification.^[^ ^44^ ^]^ As provided in the figure, the *I*_D_/*I*_G_ value of NPCSs was 0.82, which substantially increased to 0.88 in PPY(HQSA)@NPCS. The obtained data signify the successful decoration of PPY(HQSA) nanostructures onto NPCS‐GSs, which introduced additional defects to the NPCS networks. Moreover, the successful incorporation of HQSA‐dopants in the PPYs is evident by the observation of the S═O characteristic peak at 960 cm^−1^.

The specific surface area (SSA) and pore size distribution of the proposed materials were specified according to the Brunauer–Emmett–Teller (BET) method. N_2_ adsorption/desorption isotherms of NPCSs and PPY(HQSA)@NPCSs were exhibited in Figure S4a (Supporting Information); and Figure 4d, respectively. It is evident that both isotherms can be identified as type IV of IUPAC physisorption isotherms with H_4_ type of hysteresis loop. Unique porosity with a dominant portion of mesopores, in this regard, can be elucidated considering the N_2_ adsorption/desorption analysis.^[^ ^45^, ^46^ ^]^ Additionally, a well‐developed hierarchical porous structure with an average pore diameter of 4.65 and 3.5 nm was confirmed by the BJH curves of both bare and PPY(HQSA)‐decorated NPCSs, respectively (Figure S4b (Supporting Information); and Figure 4d, inset). The SSA of NPCSs and PPY(HQSA)@NPCSs were also estimated to be 1170 and 469.5 m^2^ g^−1^, respectively. Taking advantage of high SSA in parallel to the desirable pore size distribution makes bare and PPY(HQSA)‐decorated NPCSs promising candidates for energy storage applications. To gain a better insight of the structural characteristics of prepared materials, their structural features as well as their preparation condition is compared to the other commonly used methods (Table S1, Supporting Information).

**Figure 5:**
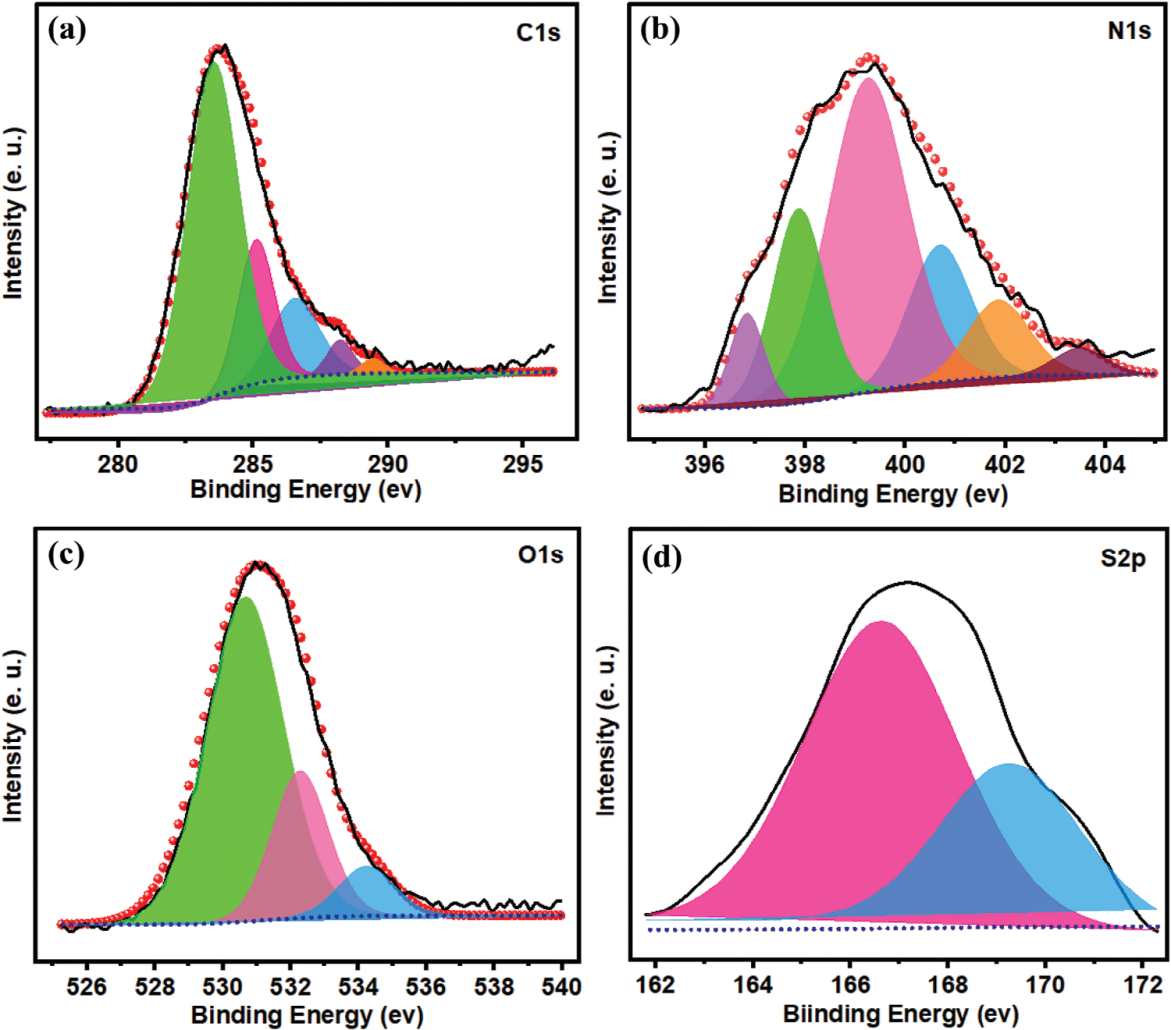
XPS deconvoluted spectra of a) C 1s. b) N 1s. c) O 1S, and d) S 2p.

Successful formation of PPY(HQSA)‐decorated NPCSs was also confirmed by X‐ray photoelectron spectroscopy (XPS). As shown in Figure S5 (Supporting Information), the observation of C, O, N, and S elemental constituents was revealed by XPS survey spectra. The deconvoluted spectrums of C1s, N1s, O1s, and S2p were further provided in **Figure** **5**a–d. As reflected in Figure 5a, the dominant peak at 283.57 eV is assigned to the sp^2^ hybridized carbon of the graphite network. The others located at 285.11, 286.5, 288.06, and 289.42 eV can be attributed to the existence of C*─*N, C*─*O/C*─*S, C═O, and O═C*─*O groups, respectively.^[^ ^47^ ^]^ Besides, the N1s peaks split into six different peaks in Figure 5b. As a direct consequence of amine groups and pyrrolic nitrogen, two substantial peaks came into view at 399.24 and 400.6 eV. Observation of these two characteristic peaks can be attributed to the successful decoration of NPCS‐GS electrodes by PPYs. Additionally, observation of two adjacent peaks at 397.87 and 396.83 eV, which are assigned to the pyridinic nitrogen and nitrile group, present credible evidence of nitrogen existence in the graphitic lattice of NPCSs. Two extra peaks at 401.85 and 403.47 eV are also attributed to the graphitic nitrogen and nitrogen oxides, respectively.^[^ ^48^, ^49^ ^]^ As represented in Figure 5c, O1s deconvoluted XPS spectrum contains three individual peaks, in which observation of a prominent peak at 530.6 eV can be ascribed to the C═O group of quinone or sulfonate fragments of HQSA dopant. Two further peaks located at 532.27 and 534.23 eV are assigned to C*─*O and O═C*─*O groups, respectively.^[^ ^50^, ^51^ ^]^ The overlapping spin–orbit doublet that was found in the S2p region implies the successful doping of HQSA into the PPY chain (Figure 5d). Two spin–orbit components, which were observed at 169 and 166.6 eV with a splitting value of 2.4 eV, are further attributed to 2p_3/2_ and 2p_1/2_, respectively.^[^ ^52^ ^]^

## 3 Electrochemical Investigations

### 3.1 Electrochemical Evaluation of Binder‐Free PPY(HQSA)@NPCS‐GS Positive Electrodes

**Figure 6:**
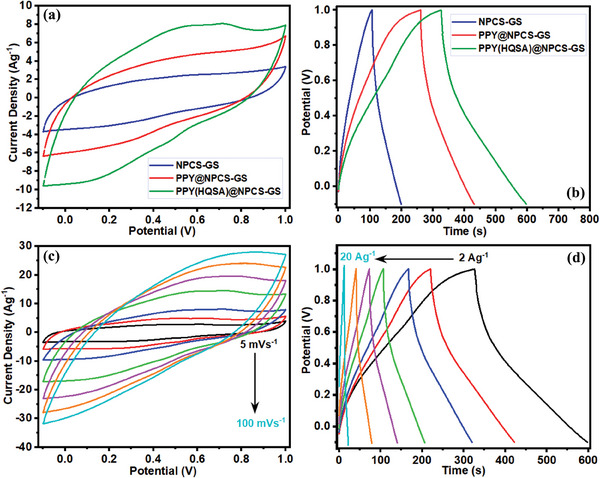
a) Comparative CVs of NPCS‐GSs, PPY@NPCS‐GSs, and PPY(HQSA)@NPCS‐GSs at 20 mV s^−1^. b) Comparative GCD curves of the prepared electrodes at 2 A g^−1^ in H_2_SO_4_ electrolyte. c) CVs of PPY(HQSA)@NPCS‐GS at different scan rates, and d) GCD curves of PPY(HQSA)@NPCS‐GS at different current densities (All measurements were conducted in 1 m H_2_SO_4_ without any redox additives).

The impact of redox‐active dopant (HQSA) on the electrochemical performance of PPY‐decorated NPCS‐GCs was studied through cyclic voltammetry (CV) and galvanostatic charge–discharge (GCD) measurements (**Figure** **6**a,b). The electrochemical performance of the fabricated PPY(HQSA)‐coated NPCS‐GSs was further evaluated in comparison to the bare bones (NPCS‐GSs). All CV measurements were conducted in a three‐electrode configuration using 1 m H_2_SO_4_ as electrolyte. For comparison, all CVs were obtained within the same potential range of −0.1 to +1.0 V versus the Ag/AgCl reference electrode. As reflected in Figure 6,a,b, upon decorating with PPYs, the electrochemical performance of NPCS‐GC electrodes enhanced dramatically. Likewise, when HQSA redox dopants were incorporated into the PPY chains, the capacitive performance of the fabricated electrodes promoted significantly. The improved electrochemical features of PPY(HQSA)@NPCSs signify the critical role of HQSA redox dopants in boosting the electric conductance of the PPYs.^[^ ^53^ ^]^ Besides, a small deviation from the rectangular shape, which appeared in the CVs of PPY(HQSA)‐decorated electrodes, can be attributed to the redox behavior of HQSA dopants. Subsequently, the electrochemical performance of the PPY(HQSA)@NPCS‐GSs was further investigated via CV and GCD measurements, conducted at various scan rates (5–100 mV s^−1^) and current densities (2–20 A g^−1^), respectively. As displayed in Figure 6c, upon increasing the potential sweep rate, no noticeable variation appeared in the CVs, revealing the appropriate capacitive performance of the prepared electrodes even in high scan rates.^[^ ^54^ ^]^ Nearly symmetric GCD curves with a negligible ohmic potential drop can be assigned to the high reversibility of charge/discharges in the H_2_SO_4_ electrolyte (Figure 6d).

To clarify the charge transfer kinetics of the prepared electrodes in H_2_SO_4_, electrochemical impedance spectroscopy (EIS) measurements were further conducted. The Nyquist plot and equivalent circuit model of PPY(HQSA)@NPCS‐GSs were provided in Figure S6 (Supporting Information). The narrow semicircle in the high‐frequency region followed by a nearly vertical line in the low‐frequency region assured the negligible charge transfer resistance (*R*_ct_ = 0.16 Ω), as well as the superior capacitive performance of PPY(HQSA)‐decorated carbon electrodes. It also concluded that the fabricated electrode exhibited a low internal resistance of 1.94 Ω. The modest internal resistance of the prepared positive electrodes can be assigned to the significant intrinsic conductivity of active materials in parallel to the reduced contact resistance of the fabricated binder‐free electrodes.^[^ ^55^ ^]^

**Figure 7:**
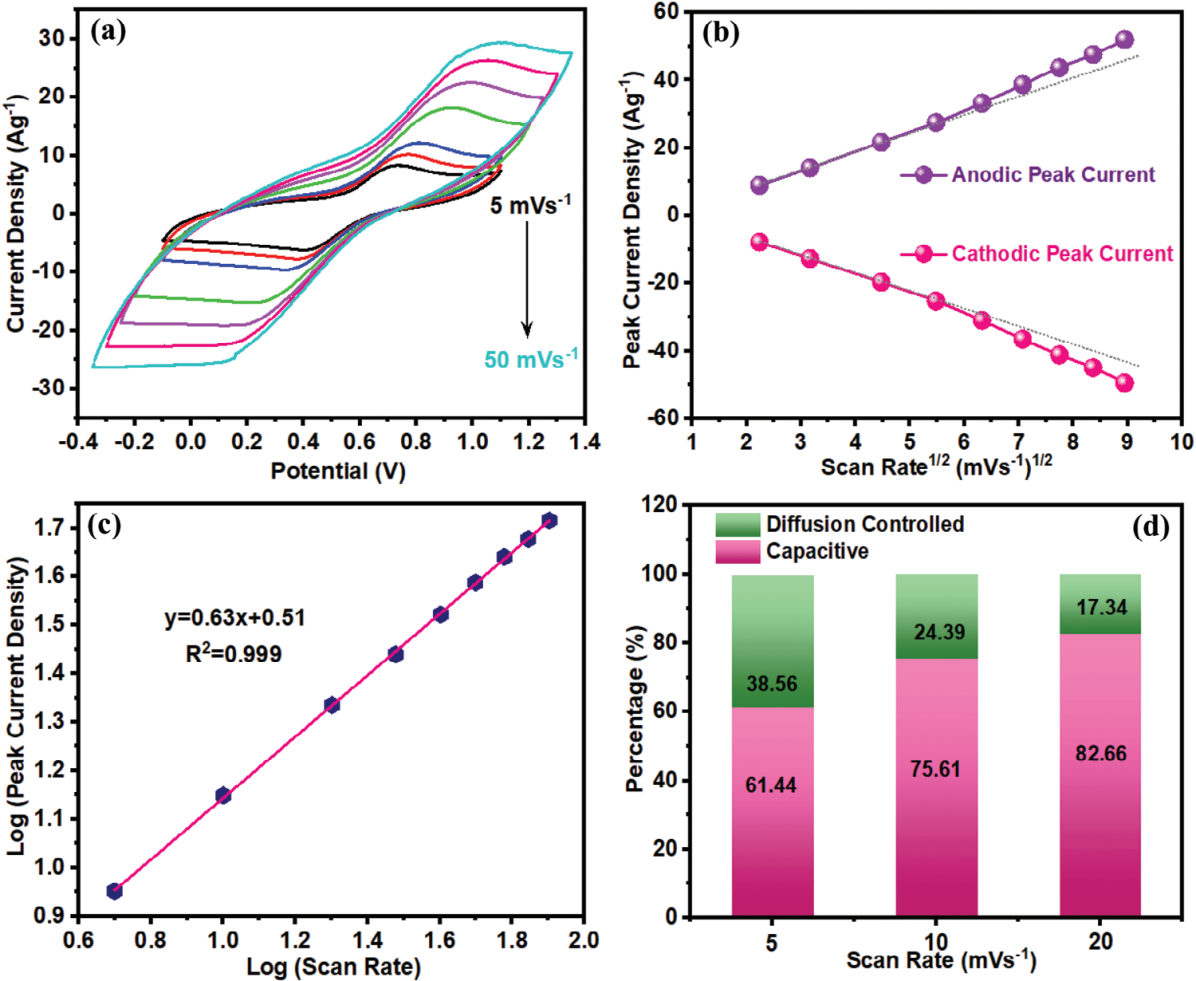
a) CVs of the PPY(HQSA)@NPCS‐GSs obtained at different scan rates in H_2_SO_4_/HQSA electrolyte. b) Peak current density as a function of the square root of scan rates. c) Log–log correlation between peak current densities and scan rates, and d) diffusion and capacitive contribution to capacitance at different scan rates.


As mentioned earlier, the energy storage performance of the PPY(HQSA)@NPCS‐GSs was further promoted by adding subsequent amounts of HQSA as redox‐active additives to form redox active electrolytes. Indeed, HQSA plays a versatile role in charge storage mechanism not only as the PPY redox dopant but also as a redox additive in hybrid electrolytes. CVs of the PPY(HQSA)@NPCS‐GS electrodes were also obtained at different scan rates (5–50 mV s^−1^) in an H_2_SO_4_ solution that contains 0.05 m HQSA (**Figure** **7**a). A pair of redox peaks that emerge in all potential scan rates mainly resulted from the following reaction:^[^ ^56^ ^]^

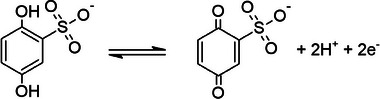
 (1)

Harnessing benefits from rapid electron transfer's kinetics, the addition of a small amount of HQSA ended in a small voltage variance between cathodic and anodic peaks at relatively low scan rates. Additionally, upon increasing the potential sweep rate, anodic, and cathodic peaks were slightly shifted to the higher potentials. These observations can be attributed to a quasireversible redox reaction of HQSA/QSA redox couples that occurred at the surface of the PPY(HQSA)@NPCS‐GS electrodes.^[^ ^57^ ^]^ Since the correlation between the peak current densities and the square root of scan rates can shed more light on the charge storage mechanism, their reliance on the charge storage was further investigated. As seen in Figure 7b, an approximately linear correlation can be assigned to the diffusion‐controlled charge storage mechanism.^[^ ^58^ ^]^ The correlation between the logarithmic values of peak current density and scan rates has been further considered for a more precise exploration of energy storage mechanisms. As displayed in Figure 7c, observation of a linear plot with a slope of 0.63 signifies the pseudocapacitive characteristic of the PPY(HQSA)@NPCS‐GSs’ energy storage in the presence of HQSA. Consequently, diffusion and surface‐controlled contributions have been examined using the following equation:^[^ ^59^ ^]^

(2) $$\frac{i\left(v\right)}{v^{1/2}}=k_{1}v^{1/2}+k_{2}$$

where *i*(*v*) and *v* refer to the current density (at a constant potential) and potential sweep rate, respectively. *k*_1_ *v* and $k_{2}v^{1/2}$ also represents the capacitive and diffusion contributions of the current. According to the linear relationship (Equation (2)), *k*_1_ and *k*_2_ can be estimated considering the slope and intercept of the linear function, respectively. As anticipated, diffusion contribution to capacitance decreases from 38.56% (at 5 mV s^−1^) to 17.34% (at 20 mV s^−1^) (Figure 7d). Conversely, surface contribution to the charge storage capacitance gradually improved with the increment of the scan rate. According to the obtained results, the precise exploration of the intercalation between HQSA and PPY(HQSA)@NPCS‐GS electrode has been provided in Figure S17 (Supporting Information).

**Figure 8:**
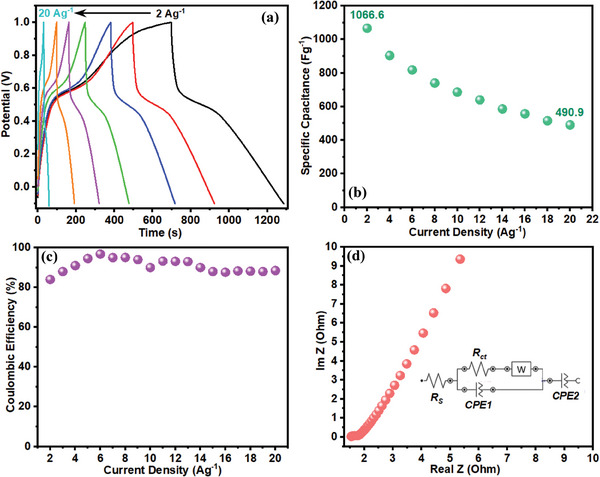
a) GCD curves of the PPY(HQSA)@NPCS‐GS at different current densities in H_2_SO_4_/HQSA hybrid electrolyte. b) Specific capacitance as a function of current density. c) Correlation between Coulombic efficiency and current density, and d) Nyquist plot and equivalent circuit model of PPY(HQSA)@NPCS‐GS in H_2_SO_4_/HQSA electrolyte.

GCD measurements of the prepared electrodes were further conducted in HQSA‐contaminated H_2_SO_4_ and the resulting curves were presented in **Figure** **8**a. The semisymmetrical GCD curves with distinct plateaus were obtained at different current densities (2–20 A g^−1^). These GCD measurements clarify the quasireversible redox reaction of PPY(HQSA)@NPCS‐GS in HQSA/H_2_SO_4_ hybrid electrolytes. Evidently, a 2.2‐fold growth in specific capacitance of the fabricated electrode (increasing from 486.3 to 1066.6 F g^−1^) is observed by introducing HQSA redox additives into the original H_2_SO_4_ electrolyte. As presented in Figure 8b, the specific capacitance of PPY(HQSA)@NPCS‐GSs moderately decreased from 1066 F g^−1^ (2 A g^−1^) to 490.9 F g^−1^ (at 20 A g^−1^), manifesting suitable rate capability of the prepared electrode, which ascribed to the hierarchical porous structure of PPY(HQSA)@NPCS active material. Moreover, a Coulombic efficiency of ≈90% in a wide range of current densities proves relatively high reversibility of HQSA redox reactions on the surface of the electrodes (Figure 8c).^[^ ^60^ ^]^ Cyclic stability of prepared positive electrode in HQSA/H_2_SO_4_ electrolyte has been evaluated during the 3000 charge/discharge cycles at current density of 6 A g^−1^. As illustrated in Figure S9 (Supporting Information), PPY(HQSA)@NPCS‐GS almost retain 85% of its initial capacitance. EIS measurements were also carried out to comprehensively assess the impact of redox additive on charge transfer kinetics of PPY(HQSA)@NPCS‐GS electrode. As displayed in Figure 8d, the observed pattern on the Nyquist plot in the presence of HQSA is like those of H_2_SO_4_ bare electrolyte. The internal resistance was significantly reduced to 1.52 Ω due to the HQSA incorporation, which is mostly attributed to the increased conductivity of H_2_SO_4_/HQSA electrolytes. On the other hand, PPY(HQSA)@NPCS‐GSs exhibited a slightly larger amount of charge transfer resistance (*R*_ct_ = 0.25 Ω) in the presence of HQSA. This can be attributed to the sluggish diffusion of organic redox species into hierarchical pore structures.^[^ ^13^ ^]^ The comparative electrochemical behavior of PPY(HQSA)@NPCS‐GS in H_2_SO_4_ and H_2_SO_4_/HQSA were also provided in Figure S7 (Supporting Information), to gain a better insight.

### 3.2 Electrochemical Investigation of Binder‐Free NPCS‐GS Negative Electrodes

**Figure 9:**
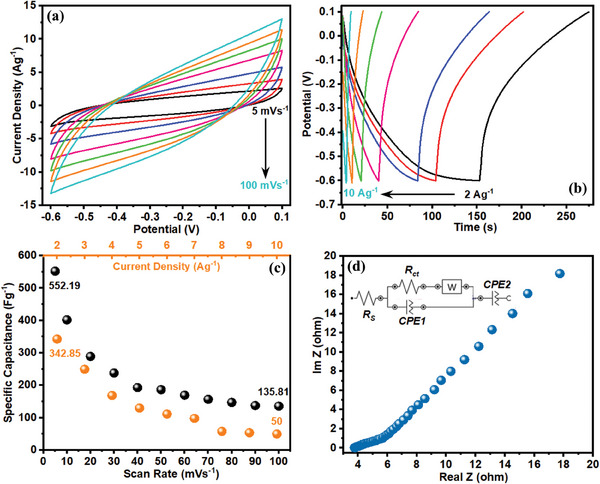
Electrochemical investigations of the NPCS‐GS negative electrodes in H_2_SO_4_ electrolyte, including a) CVs at different scan rates. b) GCD curves at different current densities. c) Specific capacitance as a function of current density and scan rate, and d) Nyquist plot and equivalent circuit model.

Being effectively involved in the overall charge storage mechanisms of asymmetric supercapacitors (ASCs), the electrochemical performance of the fabricated negative electrodes should be also investigated comprehensively. Accordingly, the electrochemical behavior of the NPCS‐GS negative electrodes was first investigated in H_2_SO_4_ electrolyte within a potential range of −0.6 to 0.1 V. As reflected in **Figure** **9**a, upon increasing the potential scan rates (5–100 mV s^−1^), the partially rectangular shape of CVs was entirely retained even at high potential scan rates. These observations ensure the ideal capacitive behavior of NPCS‐GSs. The electrochemical behavior of fabricated NPCS‐GS electrodes was further investigated using GCD measurements and the resulting GCD curves are provided in Figure 9b. As seen in the figure, the nearly symmetric triangular shape of GCD curves with negligible IR‐drop signifies that the fabricated negative electrodes store charge via a high reversibility mechanism when an aqueous solution of H_2_SO_4_ was used as electrolyte in absence of redox additives. According to Figure 9c, upon growing the potential scan rate from 5 to 100 mV s^−1^, the NPCS‐GSs experienced a diminution in specific capacitance from 552.1 to 135.81 F g^−1^. As further provided in Figure 9c, the specific capacitance of the fabricated negative electrodes decreased from 342.85 to 50 F g^−1^ by a 10‐fold increase in the current density (from 1 to 10 A g^−1^). These achievements elucidate the reasonable rate capability of NPCS‐GS in H_2_SO_4_ aqueous electrolytes. EIS measurements were also performed to achieve better insight into the charge storage mechanism of the NPCS‐GSs in H_2_SO_4_ aqueous electrolytes. As seen in Figure 9d, observation of a semicircle at high frequency region and subsequent straight line at low frequencies can be ascribed to the favorable capacitive performance of NPCS‐GS electrodes. According to the EIS outcomes, the NPCS‐GS electrodes benefit from relatively low internal and charge transfer resistances (3.77 and 1 Ω, respectively) in H_2_SO_4_ aqueous electrolytes.

**Figure 10:**
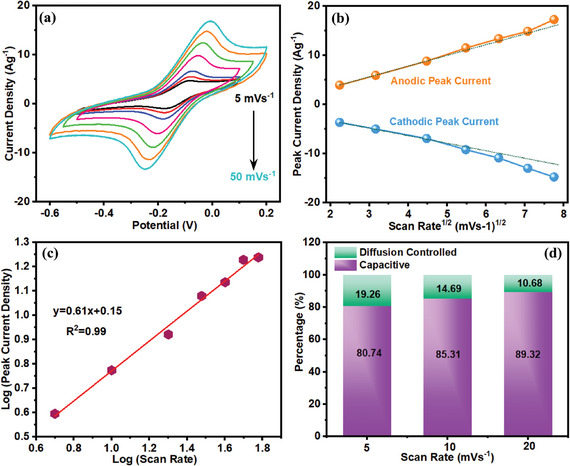
Electrochemical investigation of the NPCS‐GS negative electrodes in H_2_SO_4_/ARS, including a) CVs at different scan rates. b) Correlation between peak current densities and square root of scan rates. c) Linear relationship between logarithmic value of peak current density and scan rates. d) Diffusion and capacitive contribution to capacitance at different scan rates.


Considering the importance of negative electrodes, ARS redox additive has been also added into the bare electrolyte to specifically boost the energy storage capacitance of the adopted negative electrodes. Accordingly, the capacitive performance of the NPCS‐GSs was further assessed in 1 m H_2_SO_4_ aqueous electrolytes, containing ARS redox additives (0.03 m). As shown in **Figure** **10**a, upon increasing the potential scan rates (5–50 mV s^−1^) a pair of redox peaks came into sight in all CVs. This can be attributed to the ARS redox‐active species incorporation into the H_2_SO_4_ aqueous electrolytes. The subsequent redox reaction at the NPCS‐GS electrodes/electrolytes resulted in the observation of redox peaks within the negative potential range:^[^ ^61^ ^]^

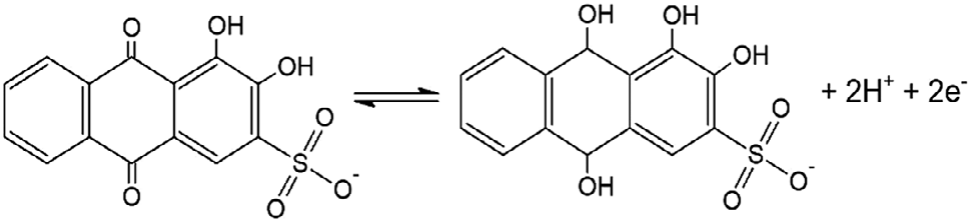
 (3)

A small potential deviation between anodic and cathodic peaks, observed in low scan rates, ensures fast charge transfer of ARS redox species, specifically in lower potential sweep rates. As reflected in the figure, this potential deviation was slightly enlarged by increasing the potential scan rates, resulting in a quasireversible redox reaction of the confined ARS at high potential sweep rates (Figure 10b).^[^ ^62^ ^]^ As depicted in the aforementioned figure, a linear relation between cathodic and anodic peak current densities and square root of scan rates provides irrefutable evidence on the diffusion‐controlled process. To have a better insight into the charge storage mechanism, the correlation between the logarithmic value of peak current densities and scan rates has been studied. As provided in Figure 10c, the slope of 0.61 reflected the pseudocapacitance energy storage mechanism of the ARS redox additives at the NPCS‐GS electrode interface. Afterward, capacitive and diffusion‐controlled contributions to charge storage capacitance have been explored using Equation (1). As seen in Figure 10d, by increasing the potential sweep rate, a stepwise growth of capacitive‐controlled contribution was observed. As seen in the figure, the greatest amount of diffusion contribution came into view in a potential scan rate of 5 mV s^−1^, where 19.26% of total capacitance originated from the diffusion process. Moreover, a capacitive contribution of 89.32% was found in the scan rate of 20 mV s^−1^. This investigation recommended that the charge storage capacitance of negative electrodes mostly resulted from the redox reactions of ARS redox species, which were confined to the porous network of the NPCS‐GSs.^[^ ^46^ ^]^

**Figure 11:**
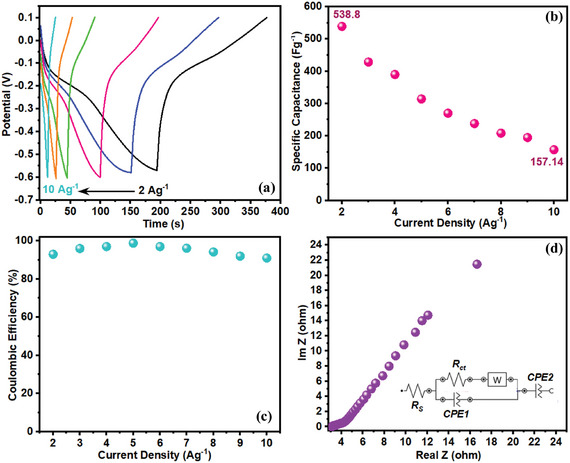
Electrochemical measurements of the NPCS‐GS in hybrid H_2_SO_4_/ARS, including a) GCD curves at different current densities. b) Specific capacitance as a function of current densities. c) Correlation between Coulombic efficiencies and current densities, and d) Nyquist plot and its corresponding equivalent circuit model.

The corresponding GCD curves of the NPCS‐GSs in H_2_SO_4_/ARS redox electrolytes were also provided in **Figure** **11**a. Enhanced discharge duration compared to H_2_SO_4_ electrolyte besides the distinct plateau of charge/discharge curves, clarify the involvement of ARS redox spices in charge storage mechanisms. Nearly symmetric GCD curves with a negligible Ohmic potential drop at different current densities (2–10 A g^−1^) reveal high reversibility of confined ARS redox reactions and good conductivity of the prepared negative electrodes. Playing a part in the charge storage mechanism, the coexist of ARS redox species in the H_2_SO_4_ aqueous electrolytes end in a significant increase in the specific capacitance of NPCS‐GS negative electrodes (almost over 1.5 times from 342.85 to 538.8 F g^−1^). As seen in Figure 11b, the specific capacitance of NPCS‐GSs in H_2_SO_4_/ARS redox electrolyte is gradually decreased from 538.8 F g^−1^ (at 2 A g^−1^) to 157.4 F g^−1^ (at 10 A g^−1^). Compared to the bare H_2_SO_4_ aqueous electrolytes, the rate capability of the NPCS‐GS negative electrodes was dramatically boosted by adding ARS redox species into the original electrolytes (1 m H_2_SO_4_). The average Coulombic efficiency of >90% can further signify the favorable reversibility of ARS redox reaction on the surface of NPCS‐GSs (Figure 11c). The energy storage mechanism of the NPCS‐GS in H_2_SO_4_/ARS redox electrolytes was further investigated using EIS technique. The corresponding Nyquist plot of the NPCS‐GSs, which exhibited the same pattern as those in H_2_SO_4_ electrolytes, manifests remarkable capacitive behavior of the prepared negative electrodes (Figure 11d). Moreover, the reduced internal resistance of the NPCS‐GSs in the hybrid redox electrolytes (*R*_s_ = 3.1 Ω) can be ascribed to the ARS redox spices contribution. In addition, the substantial decline in charge transfer resistance (*R*_ct_ = 0.5 Ω) can be also attributed to the presence of ARS redox species with reversible behavior.^[^ ^11^ ^]^ To gain a better insight into the influence of ARS presence, comparative electrochemical investigation of NPCS‐GS in H_2_SO_4_ and H_2_SO_4_/ARS were presented in Figure S8 (Supporting Information).

### 3.3 Electrochemical Investigation of Dual Redox Electrolyte Asymmetric Supercapacitors (REASCs)

**Figure 12:**
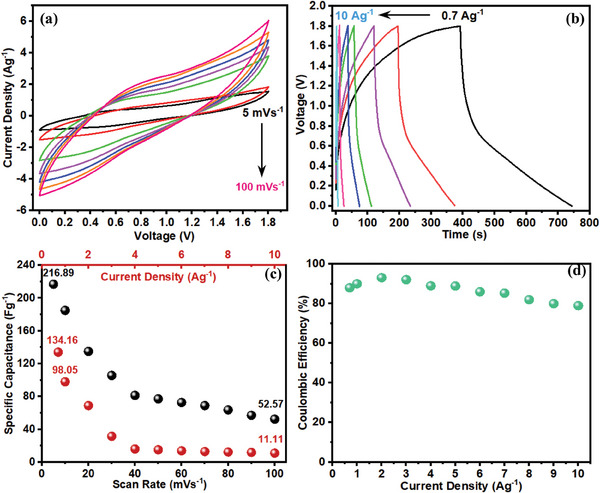
Capacitive investigations of the PPY(HQSA)@NPCS‐GS//NPCS‐GS dual REASCs, including a) CVs at different scan rates. b) GCD curves at different current densities. c) Specific capacitance as a function of current densities and scan rates. d) Relation between Coulombic efficiencies and current densities.

To validate the potential application of as‐prepared electrodes, a dual REASC device was assembled using PPY(HQSA)‐decorated and bare NPCS‐GSs as positive and negative electrodes, respectively. Besides, dual redox electrolyte systems, which were composed of 0.05 m HQSA and 0.03 m ARS were adopted for simultaneous enhancement of the electrochemical performance of both positive and negative electrodes. As seen in **Figure** **12**a, CVs of the fabricated PPY(HQSA)@NPCS‐GS//NPCS‐GS REASCs were evaluated at different potential scan rates ranging from 5 to 100 mV s^−1^. Evidently, electrochemical measurements of the prepared REASC were performed over a wide potential window of 1.8 V, which assures the high energy density of the fabricated device. Although no obvious redox peaks were found in the CVs, the slight distortion from the ideal rectangular shape reveals the pseudocapacitance mechanism in charge storage.^[^ ^1^ ^]^ Additionally, no significant variation in CVs was perceived upon increasing the potential scan rates. This can be attributed to the appropriate charge storage performance of the fabricated REASC devices even at high scan rates. As provided in Figure 12b, GCD curves of the fabricated REASCs were further studied at different current densities (0.7–10 A g^−1^). Obvious deviation from linearity observed in the GCD curves provides compelling evidence of pseudocapacitance charge storage mechanism in REASCs. Moreover, a slight IR drop in GCD curves clarifies the remarkable conductivity as well as prominent charge storage efficiency of the assembled REASC systems. Figure 12c demonstrated that the fabricated REASCs have the merit of a high specific capacitance of 134.16 F g^−1^ (at 0.7 A g^−1^). According to Figure 12d, favorable Coulombic efficiencies of the developed REASC energy storage systems (almost over 90%) assure its long‐term cycle stability.

**Figure 13:**
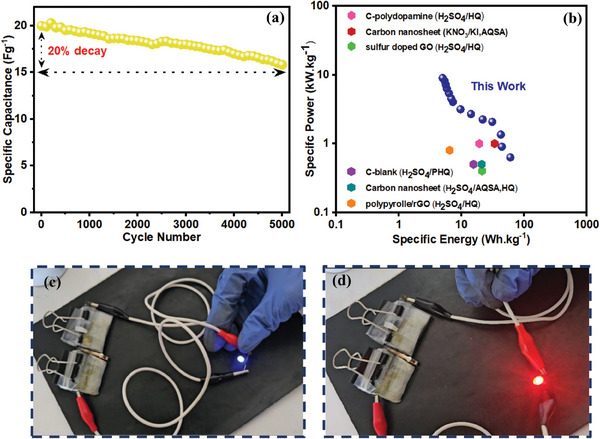
a) Cycle stability of the prepared dual REASCs. b) Ragone plot of fabricated dual REASCs.^[^ ^62^, ^63^, ^64^, ^65^, ^66^, ^67^ ^]^ c,d) Two serially connected REASCs to power red and blue LEDs.

Cycle stability of the dual redox PPY(HQSA)@NPCS‐GS//NPCS‐GS REASCs was evaluated after 5000 continuous charge/discharge cycles at a constant current density of 3.5 A g^−1^ (**Figure** **13**a). As displayed in Figure 12, the fabricated devices retained 80% of their initial capacitance after 5000 charge/discharge cycles, proving their prominent cyclability. First and last ten charge/discharge cycles were further provided in Figure S8 (Supporting Information). Taking a significant role in evaluation of energy storage systems, the Ragone plot of the fabricated devices was provided in Figure 13b. As exhibited in the figure, the fabricated dual REASCs delivered the highest energy density of 60.37 Wh kg^−1^, while taking advantage of a moderate power density of 0.63 kW kg^−1^. The maximum power density of 8.99 kW kg^−1^ was also provided in the energy density of 5 Wh kg^−1^. The charge storage performance of the assembled device also has been compared to the similar works (Table S2, Supporting Information). To reveal the practical application of the fabricated REASCs, two prepared devices were assembled in series. As demonstrated in Figure 13c,d, the serially connected REASCs were able to power red and blue light emitting diode (LED) for almost 10 and 1 min, respectively.

## 4 Conclusions

Briefly, high‐performance ASCs were assembled employing binder‐free PPY(HQSA)‐decorated and bare NPCS‐GSs as positive and negative electrodes, respectively. HQSA and ARS redox species were also utilized as cathodic and anodic redox additives. An innovative multistep procedure was presented to prepare binder‐free NPCS‐GSs. First, GSs were partially exfoliated with ultrasound waves, then roughened GSs were used as current collectors, where NPCSs were directly grown through HTC. Afterward, a creative activation method with ZnCl_2_ was applied to prepare highly porous NPCS‐GSs. Binder‐free NPCS‐GSs were then covered by PPY(HQSA) nanostructures. Utilizing dual redox electrolyte systems, containing HQSA and ARS redox species enables an extended potential window (1.8 V) in parallel to high specific capacitance (134.16 F g^−1^), assuring remarkable energy density (60.37 Wh kg^−1^) at a suitable power density of 630 W kg^−1^. The practical application of prepared REASC is also evidenced by lightening red and blue LED for minutes. The outcomes all prove that the fabricated dual redox enhanced PPY(HQSA)@NPCS‐GS//NPCS‐GS devices are applicable to large‐scale energy storage technology.

## 5 Experimental Section

Chemicals, electrodes, and device fabrication, as well as electrochemical calculations, are all provided in the Supporting Information.

## Conflict of Interest

The authors declare no conflict of interest.

## Supporting information



Supporting Information

## Data Availability

The data that support the findings of this study are available from the corresponding author upon reasonable request.
