# Supplementary material for: Revealing Energy Density in Porous Carbon Supercapacitors Using Hydroquinone Sulfonic Acid as Cathodic and Alizarin Red S as Anodic Redox Electrolytes
Source: Small. 2024 Oct 7;20(49):2406467. doi: 10.1002/smll.202406467 (PMC11618716; doi:10.1002/smll.202406467)
Supplement: Supplementary file 1 — Supporting Information [file SMLL-20-2406467-s001.docx]

**Supporting Information**

**Revealing Energy Density in Porous Carbon Supercapacitors using Hydroquinone Sulfonic Acid as Cathodic and Alizarin Red S as Anodic Redox Electrolytes**

Samaneh Abbasi ^a^, Farzaneh Hekmat ^b^, Saeed Shahrokhian ^a,^*, Mahesh Chougale,^c^ Deepak P. Dubal ^c,^*

*^a^ Department of Chemistry, Sharif University of Technology, Tehran 11155-9516, Iran*

*^b^ Department of Chemistry, Shahid Beheshti University, Tehran, Iran*

*^c^ Centre for Materials Science, School of Chemistry and Physics, Queensland University of Technology (QUT), 2 George Street, Brisbane, QLD, 4000, Australia*

S1 Chemicals

S2 Equipment and Material Characterization

S3 Preparation of Binder-Free NCS-GSs

S4 NPCS-GSs Activation

S5 Synthesis of Binder-Free PPY(HQSA)@NPCS-GSs

S6 Fabrication of REASCs

S7 Electrochemical Measurements

S8 EDS result of NPCS-GS electrode

S9 EDS result of PPY(HQSA)@NPCS-GS electrode

S10 SEM image of PPY(HQSA)@NPCS-GS after 3000 charge/discharge cycles

S11 N_2_ Adsorption-Desorption Isotherms and BJH Pore Size Distribution of NPCSs

S12 XPS survey spectra of PPY(HQSA)@NPCS-GS

S13 Nyquist Plot of PPY(HQSA)@NPCS-GSs in H_2_SO_4_ Electrolyte

S14 Comparative electrochemical performance of PPY(HQSA) in H_2_SO_4_ and H_2_SO_4_/HQSA

S15 Comparative electrochemical performance of NPCS-GS in H_2_SO4 and H_2_SO_4_/ARS

S16 Cyclic stability of PPY(HQSA)@NPCS-GS in HQSA/H_2_SO_4_ after 3000 charge/discharge cycles

S17 Assessment of the interaction between HQSA and PPY(HQSA)@NPCSs-GS in detail

S18 First and Last Ten Charge Discharge Cycles of the Fabricated Dual REASCs

S19 Structural features of prepared NPCSs compared to the other commonly used methods

S20 Comparison between this work and the other similar works

**S1 Chemical:** D (+) glucose, ammonium chloride (NH_4_Cl, 95%), acetone (99.8%), zinc chloride (ZnCl_2_), hydrochloric acid (HCl, 37%), sulfuric acid (H_2_SO_4_, 98%), hydroquinone monosulfonic acid potassium salt, alizarin red S monosodium salt was purchased from Merck and used as delivered. The deionized water (18 MΩ) of the entire process supplied by water purification system (Zolalan, Iran).

**S2 Equipment and Material Characterization:** Hydrothermal carbonization and subsequent activation process performed by laboratory oven (ACE, 400 L, Atra, Iran) and tube furnace (ATE, 1100 L, Atra, Iran), separately. Structural and chemical assessment of the prepared materials were carried out using a field-emission scanning electron microscope (FE-SEM) (TESCAN, Mira III LMU, Czech Republic), which was equipped with an energy dispersive spectroscopy (EDS) probe, transmission electron microscopy (TEM) (Philips CM200 instrument), X-ray diffraction (XRD) (X’Pert ProMPD, PANalytical, using Cu Kα radiation), Raman spectroscopy (XploRA Plus, HORIBA Ltd., Japan), and Fourier-transform infrared spectroscopy (FT-IR) ([Spectrum 100 Optica FT-IR Spectrometer, PerkinElmer](http://www.google.com/url?url=http://www.perkinelmer.com/Content/relatedmaterials/productnotes/prd_spectrum100opticaft-ir.pdf&rct=j&frm=1&q=&esrc=s&sa=U&ved=0ahUKEwigpNuGwYnaAhXkJJoKHWXcABMQFggfMAI&usg=AOvVaw10ebyWK5B7FrY4weWVuR_G), USA). The specific surface area and porous structure evaluation also conducted by Brunauer–Emmett–Teller (BET) technique at 77 K in N2 atmosphere using a surface area analyzer (Bel, Belsorp mini II, Japan).

**S3 Preparation of Binder-Free NCS-GSs:** First, a piece of GSs (2 cm × 1 cm) was exposed to ultrasound waves for 20 s in deionized water and washed with acetone several times. The pre-treated GSs were then dried at 60 °C overnight. The GSs, in turn, were slightly exfoliated with increased surface roughness. Typically, 20 mL of aqueous solution containing certain amounts of glucose (2.5 g) and NH_4_Cl at a mass ratio of 1:2 was prepared. The solution was then transferred to the Teflon-lined stainless-steel autoclave while the pre-treated GSs were soaked into the solution. The sealed autoclave was next kept at 190 °C in an electric oven for 5 h. After cooling to ambient temperature, the sample was washed with DI water several times and dried at 60 °C.

**S4 NPCS-GSs Activation:** In a typical procedure, a specific amount of ZnCl_2_ dissolved in 500 µL of DI water, in which a preferred mass ratio of ZnCl_2_ to NPCS loading was 2:1. The prepared solution drop-casted onto the covered surface of GSs, to attain ZnCl_2_ impregnated NPCS materials. The activation process was performed at 700 °C for 2h under N_2_ atmosphere. Eventually, a piece of GS, which was fully covered with NPCSs was immersed in HCl solution (3M) and maintained for 5h. This acid-treatment procedure was followed by washing with DI water and drying at 60 °C overnight.

**S5 Synthesis of Binder-Free PPY(HQSA)@NPCS-GSs:** A conventional galvanostatic electrochemical polymerization of PPY arrays was conducted employing a Metrohm Autolab PGSTAT 204 workstation. Accordingly, a piece of NPCS-GS (1 cm×1 cm) was used as the working electrode. A buffered solution at pH = 6.8, which consisted of purified pyrrole monomer and HQSA at concentrations of 0.145 and 0.010 M adopted as the deposition electrolyte. A constant current of 0.6 mA was applied for 30 min to prepare the PPY decorated NPCS-GS. A schematic illustration of the material preparation procedure is provided in Scheme 1.

**S6 Fabrication of REASCs:** In the fabrication process of REASCs, an acidic solution of H_2_SO_4_ (1 M) in coexistence with HQSA (0.05 M) and ARS (0.02 M) was used as a dual redox electrolyte. The as-prepared positive and negative electrodes at optimal mass ratio as well as the paper-based separator were soaked in the formulated dual redox electrolyte for one half hour. Afterward, REASCs were assembled using fabricated electrodes and dual redox-confined electrolytes.

**S7 Electrochemical Measurements:** Electrochemical evaluation entirely conducted with Autolab PGSTA 204 workstain (Metrohm, Netherland), which managed by NOVA (2.1) software. The graphite plate counter electrode and Ag/AgCl reference electrode were utilized in three electrode electrochemical cell. The electrochemical performance of positive (PPY@NPCS/GS) and negative (NPCS/GS) electrode in three electrode configurations were investigated in 1 M H_2_SO_4_ which separately incorporated with HQSA (0.05 M) and Alizarin red S (0.04 M). Cyclic voltammetry (CV) and galvanostatic charge/discharge (GCD) measurements were performed to extensively assessed the capacitive performance of positive and negative electrode in attendance of redox additives within the potential range of -0.1 to 1 V and -0.6 to 0.1 V, respectively. Electrochemical impedance spectroscopy (EIS) operated under the open circuit condition applying an intermittent potential of 5 mV amplitude across the potential range of 100 kHz to 0.01 Hz. Correspondingly, capacitive behavior of asymmetric dual RESCs investigated using CV and GCD technique in a dual redox electrolyte (H_2_SO_4_ in coexistence of HQSA and Alizarin red S) across the potential window of 1.8 V.

The evaluation of specific capacitance of prepared electrodes according to the CV and GCD curves are given by following equations:

$$C_{sp}=\frac{\int I\times dV}{m\times\vartheta} (S1)$$

, where $C_{\mathrm{sp}}$ (Fg^-1^) and I (A) is the specific capacitance and voltametric current, respectively.$V$ (V) is the operating potential window, m (g) is the mass loading of active materials, and $\vartheta$ is the scan rate (mVs^-1^).

$$C_{sp}=\frac{I\times\Delta t}{m\times\Delta V} (S2)$$

, in which $I$ (A) and $\Delta t$ (s) are the discharge current and the equivalent discharge time. The operating potential window and surface mass loading of active material were represented by $\Delta V$ (V) and m (g), respectively.

The specific capacitance of the fabricated REASCs is the essential value for defining the overall performance of the fabricated RESCs, which can be calculated from the following equation:

$$C_{cell}=\frac{I\times\Delta t}{M\times\Delta V} ; M=m_{anode}+m_{cathode} (S3)$$

, where Ccell (Fg^-1^), I (A), and t (s) are the cell capacitance, discharge current and the discharge time, respectively. $V$ (V) is the operating potential of the fabricated device and M (g) being the total mass loading including the mass loading of both negative and positive electrodes.

The energy and power densities of the prepared RESCs were obtained as follows:

$$E=\frac{C\times{\Delta V}^{2}}{2\times3.6} (S5)$$

$$P=\frac{E\times3.6}{\Delta t} (S6)$$

, where$E$, $C$, $\Delta V$, $P$ and $\Delta t$ are the energy density (Wh.kg^-1^), specific capacitance (Fg^-1^), operating potential window (V), power density (kW. kg^-1^) and discharge time (s), respectively.

**S8 EDS result of NPCS-GS electrode**

**Fig. S1**. EDS spectrum of NPCS-GS

**S9 EDS result of PPY(HQSA)@NPCS-GS electrode**

**Fig. S2**. EDS spectrum of PPY(HQSA)@NPCS-GS

**S 10** **SEM image of PPY(HQSA)@NPCS-GS after 3000 charge/discharge cycles**

**Fig. S3.** SEM image of PPY(HQSA)@NPCS-GS after 3000 charge/discharge cycles.

**S11 N_2_ Adsorption-Desorption Isotherms and BJH Pore Size Distribution of NPCSs**

**Fig. S4.** (a) N_2_ adsorption desorption isotherm and (b) BJH pore size distribution of NPCSs

**S12 XPS survey spectra of PPY(HQSA)@NPCS-GS**


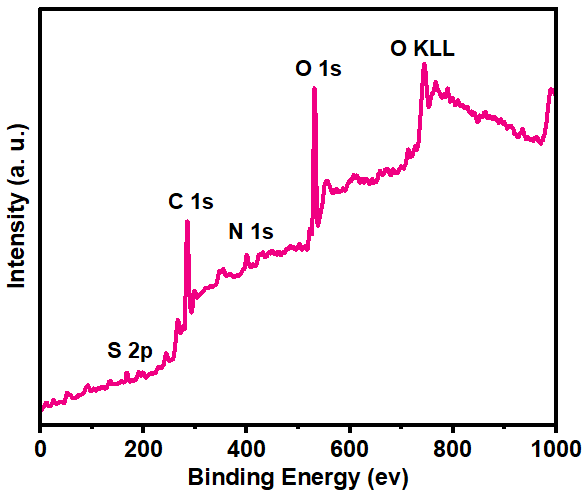


**Fig. S5.** XPS survey spectra of PPY(HQSA)@NPCS-GS

**S13 Nyquist Plot of PPY(HQSA)@NPCS-GSs in H_2_SO_4_ Electrolyte**


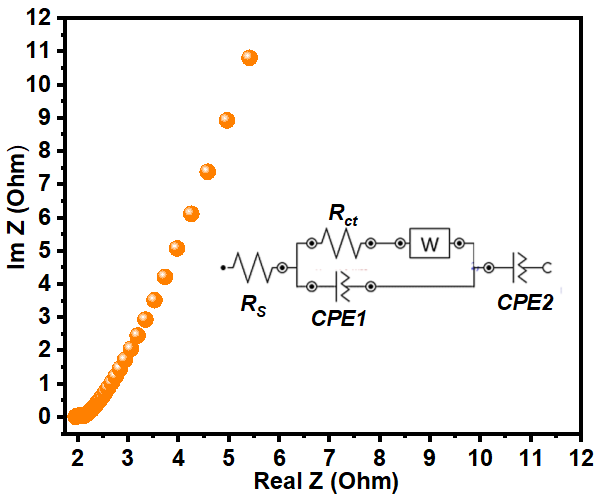


**Fig. S6.** Nyquist plot of PPY(HQSA)@NPCS-GS in H_2_SO_4_ electrolyte

**S14 Comparative electrochemical performance of PPY(HQSA) in H_2_SO_4_ and H_2_SO_4_/HQSA**

**Fig. S7.** (a) CV, (b) GCD and (c) EIS measurements of PPY(HQSA)@NPCS-GS in H_2_SO_4_ compared to HQSA contaminated H_2_SO_4_

**S15 Comparative electrochemical performance of NPCS-GS in H_2_SO4 and H_2_SO_4_/ARS**

**Fig. S8.** (a) CV, (b) GCD and (c) EIS measurements of NPCS-GS in H_2_SO_4_ compared to ARS contaminated H_2_SO_4_

**S16 Cyclic stability of PPY(HQSA)@NPCS-GS in HQSA/H_2_SO_4_ after 3000 charge/discharge cycles**


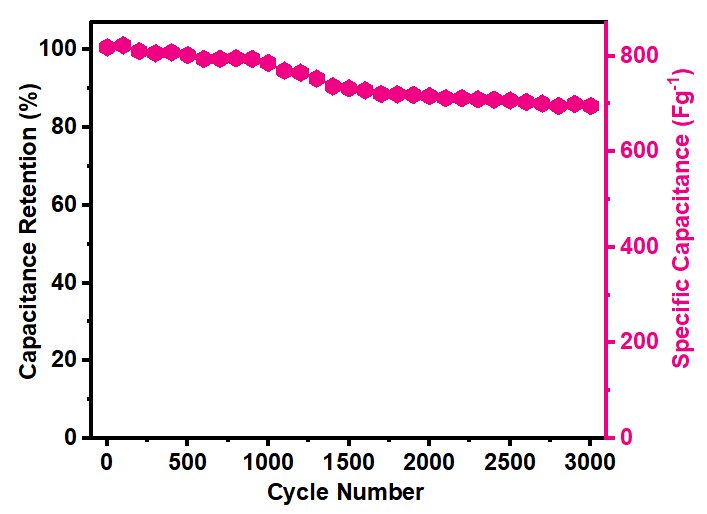


**Fig. S9.** Cyclic stability of prepared PPY(HQSA)@NPCS-GS after 3000 charge/discharge cycles in HQSA/H_2_SO_4_ electrolyte.

**S17 Assessment of the interaction between HQSA and PPY(HQSA)@NPCSs-GS in detail**

Electrochemical interaction between the PPY(HQSA)@NPCS electrode and H2SO4/HQSA electrolyte has been studied through different electrochemical measurements containing cyclic voltammetry (CV) and electrochemical impedance spectroscopy (EIS). These investigations have been described as follows:

Cyclic voltammetry (CV): To address the interaction between the redox species and active materials we should note that the most charge storage capacitance of the prepared electrode in attendance of HQSA arises from the surface-controlled reaction. More specifically, the redox species either in an oxidized (QSA) or a reduced (HQSA) state in the bulk solution is first entrapped inside the pores of the electrode, once they reach the transition states, their conversion into their adsorbed states happen. The electron-transfer reactions of these confined/adsorbed redox species ultimately lead to enhanced charge storage capacity of porous electrodes ^[1]^. Note that electron transfer reactions of redox species in transition states may also occur even without their adsorption. Although based on the given results which clarify that 61% of total charge storage capacitance is devoted to the surface-controlled reactions, the contribution of these reactions to the total capacitance is expected to be very small. More importantly, adsorbed redox species are easily converted to soluble states of O pore or R pore and subsequently diffuse to the bulk solution, resulting in short-lived charge storage processes of RESCs. Wettability and reactive sites of the active material directly affect HQSA adsorption on the surface of the electrode. In this case, various reactive sites introduced by PPY coating on the NPCS surface, efficiently enhance the redox species confinement in the pores of PPY(HQSA)@NPCS. Certainly, chemical interaction between the electrode reactive site and redox additive eases the entrapment process and enhances the energy storage capacitance of RESCs.

Electrochemical Impedance Spectroscopy (EIS): The impact of the HQSA redox additives on the charge transfer kinetics of the fabricated PPY(HQSA)@NPCS-GS electrodes were also explored through EIS measurements. The observed pattern on the Nyquist plot in the presence of HQSA is similar to those of H2SO4 bare electrolytes (in the absence of HQSA redox additives). It is clear that the internal resistance was significantly reduced to 1.52 Ω due to the HQSA incorporation, which is mostly attributed to the increased conductivity of H2SO4/HQSA electrolytes. On the other hand, a slightly larger charge transfer resistance (Rct = 0.25 Ω) was exhibited by the fabricated PPY(HQSA)@NPCS-GS electrodes in the presence of HQSA redox additives. This can be attributed to the sluggish diffusion of organic redox species into hierarchical pore structures ^[2]^. Therefore, compared to those ionic species of bare H2SO4, organic molecules of HQSA slowly diffuse into the surface of the adopted electrode and are sluggishly entrapped into the pore structure.

**S18 First and Last Ten Charge Discharge Cycles of the Fabricated Dual REASCs**

**Fig. S10.** (a) first and (b) last ten cycles of prepared dual REASC

**S19 Structural features of prepared NPCSs compared to** **the other commonly used methods*.***

**Table S1**. Structural features of the prepared NPCSs compared the other commonly used methods.

| **Carbon source** | **Molten salt or activating agent** | **Thermal treatment condition** | **Specific surface area (m^2^ g^-1^)** | **Pore characteristic** | **Ref.** |
| --- | --- | --- | --- | --- | --- |
| carrageenan | KNO_3_/LiNO_3_ | 200 °C 24h  700 °C 3h | 525.6 | mesoporous | ^[3]^ |
| aniline | NaHCO_3_ | 1100 °C 1 min  900 °C 0.5 h | 1173 | mesoporous | ^[4]^ |
| Poly(N-methylaniline) | NaCl | 1000°C 2h | 645.6 | mesoporous | ^[5]^ |
| Corn leave | LiCl/KCl | 500 °C 0.5h | 53.1 | mesoporous | ^[6]^ |
| Coal tar | NaCl/KCl | 800 °C 2h  NH_3_/Ar | 187.8 | Meso/macroporous | ^[7]^ |
| Glucose | ZnCl_2_ | 700 °C 2h | 1170 | Hierarchical mesoporous | Present work |

**S20 Comparison between this work and the other similar works**

**Table S2.** Comparison between this work and the reported similar works.

| Active material | Electrolyte additive | Power density (W kg^-1^) | Energy density (Wh kg^-1^) | Ref. |
| --- | --- | --- | --- | --- |
| C-blank | 2,5 dihydroxy benzene sulfonate | 500 | 15.6 | ^[8]^ |
| Carbon coated polydopamine | Hydroquinone | 1000 | 19.36 | ^[9]^ |
| Carbon nanosheet | KI/Anthraquinone sulfonic acid | 1000 | 33.81 | ^[10]^ |
| Sulfur doped GO | hydroquinone | 400 | 21 | ^[11]^ |
| Carbon nanosheet | Dihydroxy anthraquinone hydroquinone | 500 | 21.1 | ^[12]^ |
| PPY/rGO | hydroquinone | 80 | 6.5 | ^[13]^ |
| PPY(HQSA)@NPCSs | Hydroquinone sulfonic acid alizarin red S | 630 | 60.37 | This work |

**References:**

1. Yang, N.; Yu, S.; Zhang, W.; Cheng, H. M.; Simon, P.; Jiang, X., Adv. Mater. 2022, 34, 2202380.
2. Wang, M.; Yang, J.; Liu, S.; Che, X.; He, S.; Chen, G.; Qiu, J., Chem. Eng. J. 2023, 451, 138501.
3. Du, Y.; Fan, H.; Bai, L.; Song, J.; Jin, Y.; Liu, S.; Li, M.; Xie, X.; Liu, W., ACS Appl. Mater. . Interfaces. **2023**, 15, 4081-4091.
4. Cui, H.; Jiao, M.; Chen, Y. N.; Guo, Y.; Yang, L.; Xie, Z.; Zhou, Z.; Guo, S., Small Methods **2018**, 2, 1800144.
5. Bairi, P.; Sardar, K.; Chanda, K.; Samanta, M.; Thakur, S.; Panigrahi, K.; Sarkar, S.; Paul, T.; Chattopadhyay, K. K., ACS Appl. Energy Mater. **2020**, 3, 5984-5992.
6. Li, R.; Kamali, A. R., Chem. Engi. Sci. **2023**, 265, 118222.
7. Wei, S.; Deng, X.; Li, W.; Liu, K.; Wang, J.; Zhao, H.; Wang, X., J. Chem. Eng. **2023**, 455, 140540.
8. Zhang, Z. J.; Chen, X. Y., *Electrochim. Acta* **2018,** *282*, 563-574.
9. Zhang, Z. J.; Deng, G. L.; Huang, X.; Wang, X.; Xue, J. M.; Chen, X. Y., *Electrochim. Acta* **2020,** *339*, 135940.
10. Sun, X. N.; Xu, D.; Hu, W.; Chen, X. Y., *ACS Sustainable Chem. Eng.* **2017,** *5*, 5972.
11. Sandhiya, M.; Suresh Balaji, S.; Sathish, M., *Energy Fuels* **2020,** *34*, 11536.
12. Xu, D.; Sun, X. N.; Hu, W.; Chen, X. Y., *J. Power Sources* **2017,** *357*, 107.
13. Moyseowicz, A.; Gryglewicz, G., *Electrochim. Acta* **2020,** *354*, 136661.
